# Distinguishing Evolutionary Conservation from Derivedness

**DOI:** 10.3390/life12030440

**Published:** 2022-03-17

**Authors:** Jason Cheok Kuan Leong, Masahiro Uesaka, Naoki Irie

**Affiliations:** 1 Department of Biological Sciences, Graduate School of Science, The University of Tokyo, Tokyo 113-0033, Japan; 2 Laboratory for Evolutionary Morphology, RIKEN Center for Biosystems Dynamics Research (BDR), Kobe 650-0047, Japan; masahiro.uesaka@riken.jp; 3 Universal Biology Institute, The University of Tokyo, Tokyo 113-0033, Japan

**Keywords:** derivedness, evolutionary conservation, evo-devo, phenotypic evolution

## Abstract

While the concept of “evolutionary conservation” has enabled biologists to explain many ancestral features and traits, it has also frequently been misused to evaluate the degree of changes from a common ancestor, or “derivedness”. We propose that the distinction of these two concepts allows us to properly understand phenotypic and organismal evolution. From a methodological aspect, “conservation” mainly considers genes or traits which species have in common, while “derivedness” additionally covers those that are not commonly shared, such as novel or lost traits and genes to evaluate changes from the time of divergence from a common ancestor. Due to these differences, while conservation-oriented methods are effective in identifying ancestral features, they may be prone to underestimating the overall changes accumulated during the evolution of certain lineages. Herein, we describe our recently developed method, “transcriptomic derivedness index”, for estimating the phenotypic derivedness of embryos with a molecular approach using the whole-embryonic transcriptome as a phenotype. Although echinoderms are often considered as highly derived species, our analyses with this method showed that their embryos, at least at the transcriptomic level, may not be much more derived than those of chordates. We anticipate that the future development of derivedness-oriented methods could provide quantitative indicators for finding highly/lowly evolvable traits.

## 1. Introduction

Searching for shared features, or conserved features, among different species allows biologists to estimate a variety of ancestral features of organisms, including possible phenotypes of common ancestors, such as Urbilateria [1], their signaling pathways [2] and biomolecules [3,4]. However, when it comes to evaluating more evolved, or highly derived features or organisms, focusing only on the conserved nature may cause various inconsistencies and confusion among studies. To be specific, when trying to determine which species has phenotypically more derived features than others, comparing only conserved or commonly shared traits may underestimate how much phenotypic change has occurred since their common ancestors. This is because novel traits or lost traits are often overlooked or even ignored. For example, the pentameral symmetric body plan evolved in echinoderms is a novel trait which should not be excluded when evaluating how much the phenotypes of these species have changed since their common ancestor with other bilaterians.

This also applies to molecular-level studies. With recent increasing interest in using genome-scale omics data to study phenotypic evolution, such as the evolution of embryonic phenotypes [5,6,7,8,9,10,11], novel traits [12,13,14,15,16,17], loss of anatomical features [18,19,20], and adaptive or convergent evolution [21,22,23,24], the ambiguous use of conservation and derivedness could cause significant inconsistencies. For instance, comparisons of 1:1 orthologs tend to consider commonly shared genes among species of interest; this can be regarded as a conservation-oriented analysis. With this approach, genes that were gained or lost during evolution are often excluded. In other words, evolutionary changes achieved by genes other than 1:1 orthologs might be overlooked by these 1:1 ortholog comparisons, leading to underevaluations of how much the phenotype or the organism has derived since the common ancestor.

**Figure 1:**
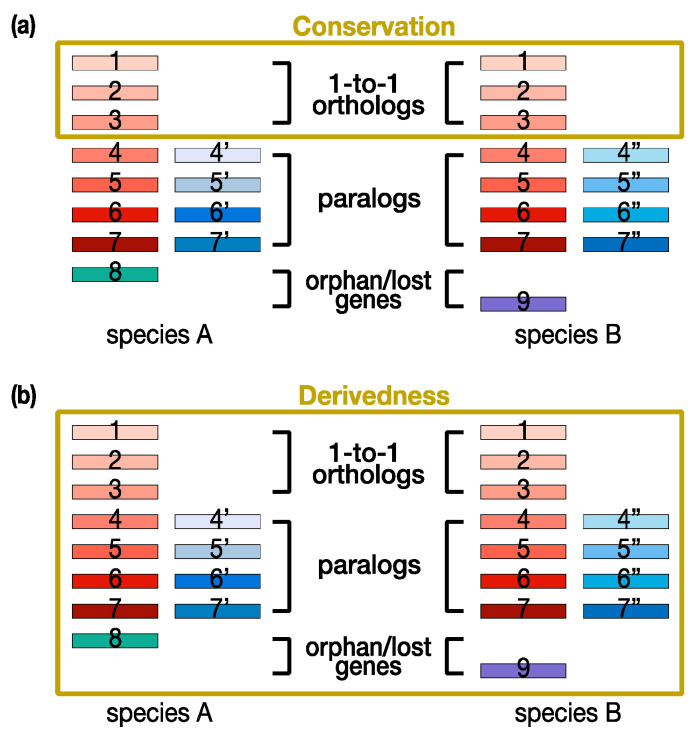
Conservation-oriented versus derivedness-oriented gene comparisons. (**a**) Conservation-oriented methods tend to compare commonly shared genes (e.g., 1:1 orthologs). (**b**) Derivedness-oriented methods additionally cover evolutionary changes of nonshared genes, such as 1-to-many orthologs, paralogs, and species-specific acquired or potentially lost genes. Rectangles: genes. Red: homologous genes inherited from the common ancestor of species A and B [27]. Blue: genes duplicated after the speciation event leading to species A and B (additionally marked by ’ and ” signs). Green and purple: orphan genes (genes without recognizable homologs in the other species).

In essence, the concept of conservation, as it has often been used in previous studies [5,11,12,25], represents information (including phenotypes and genotypes) retained during evolution. For this purpose, conservation-oriented methods tend to compare commonly shared or homologous genes and traits among the species being compared (Figure 1a). These conservation-oriented methods have been especially powerful in explaining ancestral features and species. In contrast, the concept of derivedness represents changes that have accumulated in organisms during evolution, and thus, derivedness-oriented methods tend to cover not only conserved traits and genes, but also those that were newly acquired or lost since the split from the common ancestor (Figure 1b). Because of these essential differences, it also should be noted that a less conserved feature is not always equal to a more derived one. This also applies to the relationship between being highly conserved and less derived. A possible scenario would be that if a certain organism has lost a tremendous number of traits (or genes) during its evolution, it is possible that it would be identified as an organism with highly conserved traits (or gene expression levels) by only comparing the commonly shared traits (or 1:1 orthologs). However, in this case, evolutionary changes by the loss of traits (or genes) would be overlooked, and so when such loss of traits (or genes) is also considered, it is more reasonable to consider the organism as highly derived rather than less derived.

In this review, we suggest that the differences between the two concepts, evolutionary conservation and evolutionary derivedness, and their associated methods should be handled with care; each of them has its own advantage in addressing different biological questions. We will first discuss several examples of inconsistencies which could have arisen from confusion between conservation and derivedness. We will then discuss the technical limitations and challenges of current conservation-oriented and derivedness-oriented molecular methods. Finally, as an application of the concept of derivedness, we will briefly describe our recently developed method, “transcriptomic derivedness index” [26], to estimate the evolutionary derivedness of transcriptomes of various echinoderm and chordate embryos from their common ancestor.

## 2. Confusion between Conservation and Derivedness Leads to Inconsistent Conclusions

### 2.1. Inconsistent Results Are Often Obtained from Molecular-Based Phylogenetic Estimation and Morphology-Based Estimation

**Figure 2:**
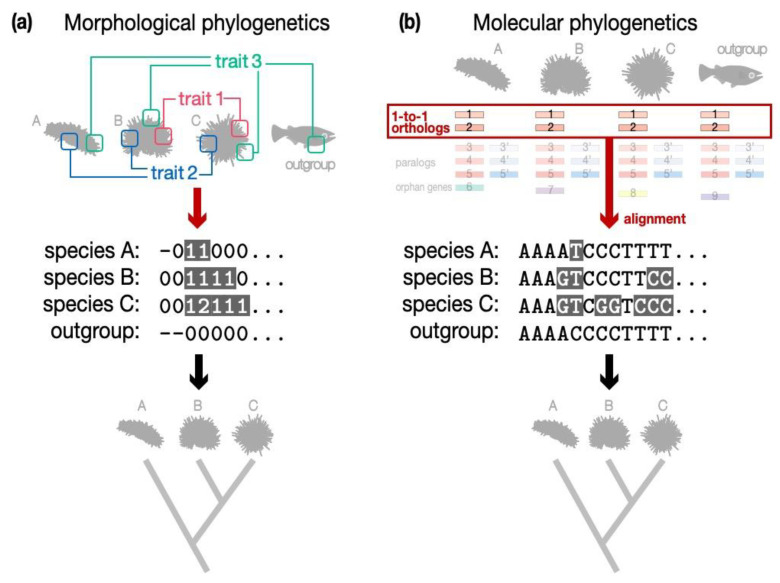
Typical methods of molecular and morphological phylogenetics. (**a**) A derivedness-oriented method, which compares not only commonly shared traits (morphological trait 3) but also those that are not shared among all species (morphological traits 1 and 2), is often used in morphological phylogenetics. For phylogenetic reconstruction, the traits of different species are encoded as different states (for example, “-” represents the absence of a trait here, “0” represents ancestral state, and “1” and “2” represent derived states). (**b**) A conservation-oriented method, which compares only commonly shared genes, is often used in molecular phylogenetics. Only the alignable sequences of the commonly shared 1:1 orthologs tend to be used to infer phylogeny.

Incongruence often appears between phylogenetic relationships estimated by a molecular and morphological approach. As a well-known example, the phylogenetic position of turtles among other vertebrates has been controversial. Many of the morphological comparisons tend to place turtles either as an early diverging lineage of reptiles or as the sister group of squamates (lizards and snakes), whereas molecular studies tend to suggest that turtles are the sister group of archosaurs (crocodiles and birds) [12,28,29,30]. This incongruence is most likely attributable to essential differences between these approaches. In short, the morphological approach tends to cover as many traits as possible (e.g., eggshell composition; Figure 2a), and evolutionary distance calculated with these traits tends to reflect the derivedness of the species, as it covers many novel and lost traits. Covering as many traits as possible is, of course, partially because there could be only a few strictly homologous morphological traits that are present in all analyzed organisms [31]. Analysis with fossil records adds further difficulty to identify commonly shared traits due to their incomplete preservation. Having said so, a major advantage of using morphological information for phylogenetics is the capability to unite extinct and extant taxa on the same tree [32], even though the approach tends to evaluate derivedness. For instance, to study early dinosaur evolution, Baron and colleagues curated the largest ever matrix comparing 457 morphological traits of dinosaurs and proposed a new hypothesis about the phylogenetic relationships of early dinosaurs [33,34,35]. This matrix included traits that are not commonly shared, as well as many missing data points due to, for example, partial fossil record.

In contrast to the derivedness-oriented morphological approach, the molecular approach tends to focus on commonly shared genomic sequences, and distances calculated with this approach tend to reflect conservation rather than derivedness. The conservation-oriented molecular methods usually align sequences of orthologous genes or their encoded amino acid sequences, followed by the reconstruction of a phylogenetic tree based on the aligned sequences (reviewed in detail in [36,37,38,39]; Figure 2b). The alignment often requires strict orthology among the genes, while paralogous genes are excluded [38]. One reason for the wide use of 1:1 orthologs is that the evolutionary model or the probability of nucleotide substitutions during evolution [40,41,42,43,44] is understood better than that of gene loss and duplications. Therefore, distinguishing true orthologs from paralogs has been a major aim of many programs in molecular phylogenetics [45]. Practically, one widely used method to identify 1:1 orthologs is to extract genes through “reciprocal BLAST best-hits”, where orthologous genes are assumed to achieve the best scores in alignments against the whole genome of another species. With the recent accumulation of whole-genome sequencing data, evolutionary analyses have switched from using only a few available genes to genome-wide comparisons. However, the basic analytical logic has not changed drastically, and commonly shared 1:1 orthologs are still preferred. For example, current popular approaches include inferring a phylogenetic tree from concatenated 1:1 orthologous genes [38]. In other words, conservation-oriented molecular methods tend to accommodate less information from paralogs and species-specific “orphan” genes (those that do not have recognizable homologous genes in other species). We note that this does not mean that these often excluded genes do not convey any phylogenetic information, as a recent study suggested that paralogous genes may also have the potential for phylogenetic reconstruction [46].

Therefore, in addition to potential impact from missing data [47] and evolutionary events such as convergent evolution [48] that could mask the true phylogeny, phylogenies estimated by the conservation-oriented and derivedness-oriented approach could possibly be inconsistent.

### 2.2. Conservation-Oriented Approach May Be Less Sufficient in Elucidating Derived Features

We next discuss the potential insufficiency in understanding derived traits and species if only conservation-oriented methods are deployed. Especially, arguments over the evolutionary rate often become confusing or seemingly contradictory when conservation and derivedness are mixed up. One good example would be the discussion on the evolutionary rate of species such as “living fossils”. These species, such as the coelacanth and the tuatara, retain many ancestral features and remain morphologically similar to their fossil relatives that existed millions of years ago. In other words, their overall rates of morphological changes tend to be slow, and it may be more suitable to describe them as less derived species rather than highly conserved species. Seemingly consistent with this, the genomic evolutionary rates of these species estimated on the basis of conserved 1:1 orthologous genes are reportedly slower than those of their sister groups [49,50]. However, occasional counterarguments to this view [51,52] point out that studies which demonstrated rapid molecular evolution in the coelacanth, especially those focused on specific gene sets, are often neglected. Such conflict could potentially arise because these molecular arguments tend to be based on commonly shared genes, or conservation-oriented evaluations while the former standpoint is focusing more on derivedness-oriented features. To be specific, an example is the analysis performed by Cavin and Guinot [53], which showed that the coelacanth acquired much fewer autapomorphies (new derived traits) per million years during its evolution than two other crown-group vertebrate species. Thus, living fossil species are expected to show low phenotypic derivedness as a whole (also considering the nonshared traits [53]), but this does not necessarily mean that the rate of sequence evolution (estimated with conservation-oriented methods for a limited set of genomic sequences) must also be slow. To add, this cannot be fully explained by a gap between genotype and phenotype. Even if we could measure evolutionary rate of all the conserved traits that are shared in species being compared, it would be insufficient to evaluate if the species acquired fewer novel traits or lost fewer traits to remain less derived.

## 3. Technical Limitations of Current Conservation-Oriented and Derivedness-Oriented Molecular Approaches

Although both conservation-oriented and derivedness-oriented approaches have different strengths in understanding evolution, each approach has various limitations, and thus methodology should be carefully selected depending on the purpose of research.

The conservation-oriented molecular comparisons tend to rely on commonly shared genes (e.g., 1:1 orthologs), and this approach has a limitation when a large number of species are analyzed. To be specific, due to the strict definition of 1:1 orthologs, these genes often cover only a small part of the entire gene repertoire in the genome, especially when a large number of species are compared. For example, in our recent analysis comparing 13 species of chordates and echinoderms [26], we identified only 271 1:1 orthologous genes, which covered only ~1.5% of all the genes in a typical deuterostome genome (~20,000 genes). While these 1:1 orthologs would still be sufficient for reconstructing phylogeny [38] or estimating the evolutionary rate of each species, their analysis may not be sufficient for evaluating derived features, or changes that accumulated during evolution, as it ignores changes that took place in the remaining ~98.5% of the gene repertoire. Even when a more sophisticated search method was employed, only 1126 1:1 orthologs were found in 18 metazoan species [54]. As a result, the evolutionary changes within more than 90% of the genes will be excluded. Thus, the conservation-oriented molecular approach may significantly underestimate evolutionary changes, especially when derivedness is in focus. Consequently, the conservation-oriented approach may be less sufficient to elucidate the complete molecular mechanism of how derived phenotypic traits emerged or evolved. One good example would be a study done by Gildor and colleagues [11]. To understand how changes in developmental gene expressions alter morphogenesis of echinoderm species, they analyzed the gene expression profiles of 8735 1:1 orthologs among three echinoderm species (a sea star and two sea urchins), and these 1:1 orthologs correspond to only around half of the entire gene repertoire of each species. This conservation-oriented approach was sufficient to identify conserved developmental stages (such as the gastrula stage) among the three echinoderm species. However, as the authors argued, they pointed out that their analysis might have underestimated the differences of expressions of nonshared genes (such as genes that are important for skeletogenesis in sea urchins but are absent from the sea star genome). And they also argued that these overlooked genes might contribute to species-specific, or at least sea urchin-specific, morphological features. A similar discussion was made in the study trying to elucidate the evolution of an elaborated structure called the “helmet” in treehoppers. By comparing the transcriptomic profiles of 1:1 orthologs between treehoppers and leafhoppers (their sister group which retains an ancestral condition in the dorsal wall), Fisher and colleagues [15] found that the elaborated helmet may have evolved through coopting the wing-patterning network from the common ancestor of treehoppers and leafhoppers. Using this conservation-oriented approach, many commonly shared genes in the wing-patterning network could be identified as being expressed in the elaborated helmet, supporting the co-option hypothesis. However, this has yet to explain why the coopted gene expressions could transform the dorsal wall into a much more elaborated helmet morphology in treehoppers. Further studies are awaited to fully elucidate the molecular mechanism, and it is tempting to know if derivedness-oriented approach encompassing the nonshared genes would yield additional insights. As in the other studies, 7635 1:1 orthologs could be identified from the de novo assembled transcriptomes of the two species, which only corresponds to approximately half of the entire gene repertoire.

In contrast to the conservation-oriented approach, evolutionary changes achieved by duplicated, newly acquired and lost genes could play significant roles in phenotypic evolution because they are often considered as important drivers of phenotypic innovations [55,56,57]. However, despite the possibility to include these nonshared genes into comparison, current derivedness-oriented approaches also have a major limitation. There is still no widely accepted way to compare the attributes (such as expression levels) of paralogs, newly acquired genes, species-specific orphan genes, and potentially lost genes across species, although several trials have attempted to compare them using an ortholog-group approach [7,58]. In Levin et al. [58], the expression level of an ortholog-group was determined by the expression level of the gene with the largest expression fold change, although they found no significant difference when randomly selecting a paralogous gene as the representative expression level of the ortholog-group. Similarly, in Hu et al. [7], the authors found essentially the same results (i.e., persistent conservation of the mid-embryonic body plan-developing phase in vertebrates) by taking either the mean or the sum expression level of all predicted paralogous genes to define the expression level of an ortholog-group. These approaches are mostly based on the assumption that the functions of the putative ancestral gene became distributed among paralogs while neofunctionalization could occur, and yet further research is needed to assess to what extent these estimation methods are appropriate, or whether more sophisticated models of transcriptome evolution should be incorporated into the estimation (such as insights from studies that aimed to investigate the evolution of gene expressions involving duplicated genes [59,60]). For other potential weak points of the derivedness-oriented molecular approach, we will further discuss them in the next section.

Lastly, we note that although we discussed comparing 1:1 orthologs in comparative transcriptomic studies tend to be a conservation-oriented method, it does not mean that comparing 1:1 orthologs must always be conservation-oriented. A possible exception is that when two organisms being compared have the exact same pairs of orthologs, comparison of 1:1 orthologs could be regarded as both conservation-oriented and derivedness-oriented method. Hence, methodologies are not always linked with either conservation-oriented or derivedness-oriented, and analytical methods and data should be designed to fit the purpose of research.

## 4. “Transcriptomic Derivedness Index” for Quantifying Degree of Phenotypic Evolution of Embryos

In the last decade, the field of evolutionary biology field has shown increased interest in the use of molecular data to study phenotypic evolution, as in the examples discussed above. However, without further development of derivedness-oriented methods, the evolutionary changes that were achieved by genes other than 1:1 orthologs could be largely underestimated or even overlooked.

**Figure 3:**
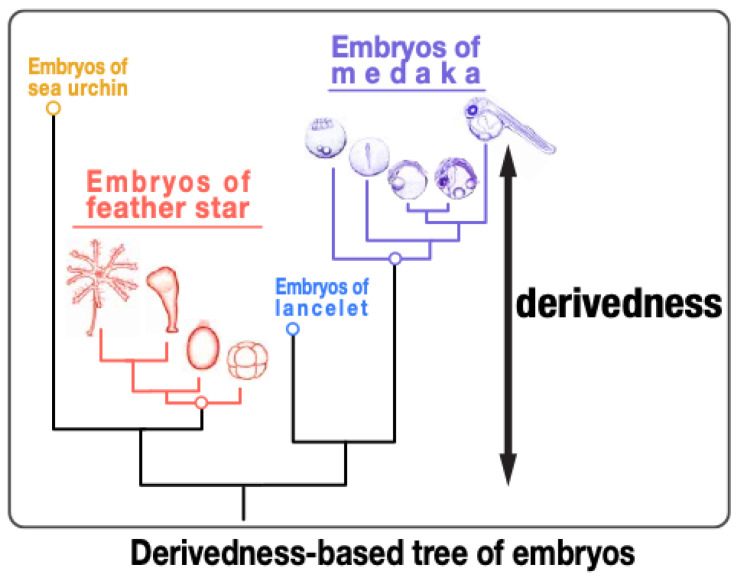
Quantification of phenotypic derivedness of embryos. The whole-embryonic transcriptome was utilized as a phenotype, and we developed a method to estimate the transcriptomic derivedness of each developmental stage from the common ancestor of echinoderms and chordates. In addition to 1:1 orthologs, expression levels of paralogs and potentially lost genes were also considered when calculating the evolutionary distances between embryonic transcriptomes. Transcriptomic derivedness of each developmental stage was then plotted as the total branch length from the common ancestral node on the inferred tree (modified from [26]).

In this respect, we recently attempted to develop a method to quantify the degree of phenotypic evolution of embryos, or degree of their derivedness, by incorporating the expression profiles of paralogous genes (where we utilized the transcriptome as a phenotype of an embryo) [26]. We focused on echinoderms, a group of animals that are generally considered as highly derived species on the basis of their unique anatomical features, such as pentameral symmetry in the adult stage [61,62]. Meanwhile, it remains to be tested whether their molecular developmental systems, especially their pentameral developmental stages, are also much more derived than the bilateral embryonic stages. Similarly, although the evolutionary conservation of the mid-embryonic period has been clarified in a wide variety of animals (including vertebrates [5,6,12,63,64,65], *Drosophila* species [66,67], nematodes [68], and mollusks [69]), this does not necessarily mean that this embryonic period remains less derived than the earlier and the later stages because the nonshared genes have not been considered. In our recent study [26], we compared the expression profiles of not only 1:1 orthologs, but also paralogs and potentially lost genes of various echinoderm and chordate species to quantify the evolutionary derivedness of their developmental stages (Figure 3). Data were previously collected from wild-type embryos, with biological replicates from independent parents in order to represent the statistical population of interest by incorporating phenotypic variations among the wild-type embryos.

To mitigate the limitation imposed by conventional conservation-oriented methods, we proposed that transcriptomic derivedness could be better estimated by comparing the expression profiles of ortholog-groups instead of 1:1 orthologs, taking references from the methods used in some pioneering studies [7,58]. To identify the genes of ortholog-groups encompassing distantly related species, we compared protein-coding genes, and identified ortholog-groups using the PORTHOMCL software (which clusters genes on the basis of sequence alignment results from BLASTP) [70,71]. These ortholog-groups also include paralogs, acquired and potential lost genes, so their expression changes could also be evaluated. For the 13 species, 22,689 ortholog-groups were identified, covering an average of 76% of the entire gene repertoire in each species. Meanwhile, only 271 1:1 orthologs could be identified from reciprocal BLAST best-hits. We note that although this approach allowed the comparison using ortholog-groups, the ortholog-prediction software may have limited power to distinguish genes newly acquired in certain lineages and genes lost in other species. In comparing the expression levels of ortholog-groups, the expression level of a certain ortholog-group was calculated as the mean expression value of the constituent paralogous genes (taking the sum expression value yielded similar results). For those species that lack any homologs of the ortholog-group (i.e., potentially lost genes), an expression level of 0 was assigned. We note that the ortholog-group prediction approach that we utilized may have a limited power to distinguish between newly acquired genes and genes potentially lost in other lineages, but it is reasonable to assign 0 expression levels to these potentially lost genes because they are not expressed. A method suitable for calculating evolutionary distance based on the expression profiles of ortholog-groups was defined by the criteria including: developmental stages cluster by species on the inferred tree of embryonic transcriptomes, and the topology of this tree is consistent with the phylogeny estimated from genomic sequences (as in Figure 3). However, the tree covering orphan genes (genes that have no homologs in the other species) violated the second criterion. Therefore, to be conservative, in further analyses in the study, we adhered to the method and the tree that showed a topology consistent with the phylogeny estimated from genomic sequences, where only 1:1 orthologs, paralogs and potentially lost genes were considered in the calculation (discussed in detail in [26]). We note that it is also possible that the derivedness tree does not strictly match the phylogenetic relationship estimated from genomic sequences, compromising the second criterion. For instance, if the phenotype of a species (including the developmental transcriptomes) evolves extraordinarily rapidly, the phenotypic derivedness tree topology may be inconsistent with that estimated from genomic sequences. Moreover, phylogeny estimated by genomic sequences mostly rely on alignable sequences across species (as explained briefly in Section 2.1) where species-specific orphan genes are excluded. Besides these, accurate estimation of evolutionary changes due to orphan genes could be challenging, because they could be misannotated as they have no homologous counterparts in other species. In *Xenopus laevis* (African clawed frog), ~40% (7879 genes) of its predicted orphan genes are expressed at low or undetectable levels in all developmental stages examined, which could imply misannotation [26]. Therefore, although our proposed method has greatly increased the number of genes that could be compared among species and allowed us to evaluate the transcriptomic derivedness of embryos, this method is still unable to cover evolutionary changes caused by species-specific orphan genes, and further research is required to better accommodate these genes into the estimation of the transcriptomic derivedness of embryos.

Finally, using this ortholog-group approach, irrespective of whether the species-specific orphan genes were included or not, we surprisingly found that the embryonic transcriptomes of echinoderm species as a whole may not be much more derived than those of chordates since their common ancestor, but the pentameral phase of echinoderms indeed shows higher transcriptomic derivedness than the bilateral phase. In vertebrate species, we found that the conserved mid-embryonic period is also less derived compared to the earlier or the later developmental stages. We further sought to ask which orthologous gene groups could potentially explain the higher or lower derivedness of these developmental stages of interest. Our analysis showed that the higher derivedness of the pentameral phase of the green sea urchin appears to be attributable to genes being expressed at different levels in the pentameral phase than in the bilateral phase, rather than having different sets of genes being expressed in different phases. Meanwhile, the lower derivedness of the vertebrate mid-embryonic period is correlated with the expression of many genes involved in organogenesis such as the *Hox* ortholog-groups. Interestingly, different functional subsets of ortholog-groups may have different explanatory power in addressing the differences in transcriptomic derivedness index across species. For example, the embryonic transcriptomes of the green sea urchin tended to show higher derivedness than those of the purple sea urchin, but their overall difference was reduced in a tree drawn by ortholog-groups consisting only of transcription factors.

One surprising, or perplexing finding from our study [26] was that the embryos of the tunicate species, *Ciona intestinalis*, showed lower transcriptomic derivedness than those of the other chordate species despite the rapid evolutionary rate of the *C. intestinalis* genome [72,73]. A possible reason for this inconsistency would be that, similar to what we discussed in Section 2 of this review, estimation of evolutionary rate is mostly evaluated using commonly shared genes whereas the estimation of transcriptomic derivedness additionally covers nonshared genes. This could be further complicated by the inclusion of a large number of potentially lost genes in *C. intestinalis* as the effect of assigning 0-expression levels to potentially lost genes remains to be explored. Therefore, it may be interesting to further include developmental transcriptomic datasets of other species, such as the appendicularian tunicate *Oikopleura dioica* which is considered to have lost many essential genes, to investigate whether low transcriptomic derivedness is general in tunicates or not, and to study potential technical biases related to potentially lost genes. Importantly, while our method could be more suitable for evaluating derived features of embryos than merely comparing expression levels of 1:1 orthologs, further research is necessary to take into account technical concerns such as: (i) the bias from potential differences in read depth among RNA-seq samples, which may affect the measured expression levels, (ii) difference in the quality of genomes and annotations, which may affect ortholog-group prediction, and (iii) the effect of assigning 0-expression levels to potentially lost genes. We also found a moderately strong correlation between the transcriptomic derivedness index and the number of predicted ortholog-groups (i.e., more ortholog-groups could be identified for vertebrate and sea urchin species, and their median derivedness indices tend to be higher); however, the reason for this correlation and the extent to which the expression levels of ortholog-groups contributed to their higher derivedness remain unknown; however, the reason for this correlation and the extent to which the expression levels of ortholog-groups contributed to their higher derivedness remain unknown. Future studies are needed to assess the potential influence of the number of ortholog-groups being compared. Finally, when these technical issues are more thoroughly investigated, it would be intriguing to apply this method to study the derivedness of species in other phyla and other phenotypic traits as more data of other lineages of interest continue to accumulate. To add, to more comprehensively evaluate phenotypic derivedness of embryos or species, other phenotypic traits besides the transcriptome should ideally be considered as well.

## 5. Conclusions

To conclude, herein, we discussed the differences between the concepts of conservation and derivedness: conservation mainly represents information retained during evolution, whereas derivedness represents changes during evolution; this difference should be dealt with carefully. Accordingly, evaluations of conserved information are not equivalent to evaluations that include derived or nonshared traits, and confusion between the two could lead to various problems such as inconsistencies between phylogenies estimated by molecular and morphological approaches, and confusing arguments over the evolutionary rates of living fossil species.

In this respect, it is important to carefully select the appropriate method depending on the purpose of research. We discussed the notion that ancestral, shared features could be more easily identified by conservation-oriented methods, while derivedness-oriented methods could be more sufficient in addressing evolutionary changes that were achieved not only by modifications in conserved genes, but also by gene duplication, acquisition, or loss. Technically, when analyzing genome-scale data, 1:1 orthologous genes may cover only a small proportion (~1%) of the entire genome when many species are compared, so conservation-oriented methods may unintentionally discard much of the information which is important for the analysis of phenotypic evolution. However, derivedness-oriented methods are currently not as well developed as conservation-oriented ones. Although the ortholog-group approach has been used in several studies [7,26,58], the question of how to compare the expression levels of ortholog-groups by covering paralogs, acquired genes, and potentially lost genes may still require further investigation. Well-developed evolutionary models for novel and potentially lost traits and genes are also needed. We anticipate that further studies in these aspects will facilitate future development of derivedness-oriented methods.

Finally, considering that phenotypic evolvability addresses phenotypic changeability during evolution, it is expected that highly evolvable features would be, at least, highly derived. Given this, although derivedness itself does not directly reflect evolvability, a potential future application of derivedness-oriented methods would be to find candidate highly evolvable traits or biological processes (see [1] for a more detailed discussion related to the implications of our recently developed method, the “transcriptomic derivedness index”).
